# The C‐terminal extension of calprotectin mediates zinc chelation and modulates *Staphylococcus aureus* biomass accumulation

**DOI:** 10.1002/pro.70294

**Published:** 2025-09-13

**Authors:** Yasiru R. Perera, Kyle T. Enriquez, Aslin Rodriguez, Velia Garcia, Tae Akizuki, Anais Naretto, Melumo Togashi, Ryan Guillen, Eric P. Skaar, Walter J. Chazin

**Affiliations:** ^1^ Departments of Biochemistry and Chemistry Vanderbilt University Nashville Tennessee USA; ^2^ Center for Structural Biology Vanderbilt University Nashville Tennessee USA; ^3^ Vanderbilt University Medical Scientist Training Program Vanderbilt University School of Medicine Nashville Tennessee USA; ^4^ Department of Pathology, Microbiology, and Immunology Vanderbilt University Nashville Tennessee USA; ^5^ Vanderbilt Institute for Infection, Immunology, and Inflammation Vanderbilt University Medical Center Nashville Tennessee USA

**Keywords:** biofilm, calprotectin, nutritional immunity, *Staphylococcus aureus*, X‐ray crystallography, zinc

## Abstract

Calprotectin (CP) is an S100A8/S100A9 heterodimer that plays an important role in nutritional immunity at the host–microbe interface. CP combats *Staphylococcus aureus* growth by sequestration of zinc and other trace transition metals; however, questions remain about whether CP antimicrobial activity strictly relies on metal sequestration. Moreover, the precise mechanism for how zinc binds at the two distinct transition metal binding sites of CP is not known. High‐resolution X‐ray crystal structures reveal tetracoordinate binding in the canonical His_3_Asp site and hexacoordinate binding in the His_6_ site similar to the binding of manganese and nickel in this site. The S100A9 C‐terminal extension (tail) contributes two of the His residues in the His_6_ metal‐binding site, but measurements of zinc affinity show there is no significant reduction upon mutation of these His residues or deletion of the entire C‐terminal tail. Bacterial growth and static biofilm assays show that the His mutations affect *S. aureus* biomass accumulation differently than loss of the S100A9 C‐terminal tail, despite resulting in the same defect in bacterial–CP binding. These results reveal that the S100A9 tail of CP has a role in preventing *S. aureus* biomass accumulation.

## 1 INTRODUCTION

*Staphylococcus aureus* is opportunistic, Gram‐positive, adherent bacteria that include methicillin‐susceptible and methicillin‐resistant strains (MSSA and MRSA). This pathogen is a predominant cause of bacterial‐derived morbidity and mortality worldwide (Ikuta et al. 2022; Murray et al. 2022). *Staphylococcus aureus* contributes to skin and soft tissue, vascular, cardiac, bone, prosthetic joint, and numerous other infections (Liu et al. 2011). *Staphylococcus aureus* aggregate into multicellular structures, termed biofilms, via a regulated process of adhesion, growth, maturation, and dispersal (Cruz et al. 2018; Flemming and Wingender 2010; Kaplan et al. 2012; Macia et al. 2014; Toledo‐Silva et al. 2021). Epidemiology suggests that *S. aureus* biofilms significantly contribute to pathogenesis of clinical infection as well as development of increasing resistance to antibiotics and host immune responses (Otto 2008; See et al. 2020). Staphylococcal biofilms and bacterial aggregates are composed of extracellular polymers including nucleic acids, extracellular proteins, and polysaccharides that limit bacterial surface exposure to environmental stress (Abriat et al. 2020; Flemming and Wingender 2010; Jeffries et al. 2021; Kaplan et al. 2012; Romaniuk and Cegelski 2015; Thongsomboon et al. 2020). Once formed, they confer resistance of the community to desiccation, antimicrobials, shearing forces, and other environmental stressors (Abriat et al. 2020; Cruz et al. 2018; Flemming and Wingender 2010; Haque et al. 2021; Kaplan et al. 2012; Macia et al. 2014; Romaniuk and Cegelski 2015; Syed et al. 2021; Toledo‐Silva et al. 2021).

A key role has been established for zinc ions (Zn^2+^) and other trace first‐row transition metals in staphylococcal biology and pathogenesis (Cassat and Skaar 2012; Hood and Skaar 2012; Murdoch and Skaar 2022; Rehder et al. 2015). It is therefore unsurprising that sequestration of these nutrient metals by innate immune factors plays a critical role in nutritional immunity at the host‐microbe interface (Hood and Skaar 2012; Zackular et al. 2015; Zygiel and Nolan 2018). The S100A8/S100A9 heterodimer termed calprotectin (CP), which comprises >40% of the cytosolic content of neutrophils, is one such factor. CP sequesters zinc and other trace transition metals and serves as a key mediator of nutritional immunity (Brophy and Nolan 2015; Cho et al. 2015; Damo et al. 2013; Kehl‐Fie et al. 2011; Wakeman et al. 2016; Zygiel et al. 2021). The loss of CP is detrimental for the host in combating infection caused by numerous pathogens, including *S. aureus* (Edgeworth et al. 1991; Tardif et al. 2015).

S100 proteins, including CP, form obligate dimers and many contain symmetrically disposed tetrahedral transition metal binding sites at the interface between the two subunits. Binding of Zn^2+^ and Cu^2+^at these sites is well established. CP contains one canonical tetrahedral His_3_Asp transition metal binding site formed by the side chains of His83 and His87 from S100A8 and His20 and Asp30 from S100A9. However, CP is unique in its ability to bind a wider range of transition metals with very high affinities, a consequence of its distinct octahedral binding site comprised of six histidine side chains: His17 and His27 from S100A8 and His91, His95, His103, and His105 from S100A9 (His_6_ site) (Damo et al. 2013; Nakashige et al. 2018). While both sites bind Zn^2+^ and copper with high affinity, the hexacoordinate His_6_ site also binds manganese (Mn^2+^), nickel (Ni^2+^), and iron (Fe^2+^) ions with high affinity (Damo et al. 2013; Nakashige et al. 2018). The His_6_ site is a by‐product of the C‐terminal extension in S100A9 relative to other S100 proteins. This extension, referred to as the C‐terminal tail, contributes two histidine residues (His103 and His105) to the His_6_ site. Beyond the unique transition metal binding site, the C‐terminal tail has been implicated in a range of additional functions, including binding and transport of fatty acids (Klempt et al. 1997). Knowledge of the binding of transition metals to CP is based on extensive biochemical characterization and crystal structures with Mn^2+^ or Ni^2+^ in the His_6_ binding site (Baishya et al. 2022; Besold et al. 2018; Corbin et al. 2008; Damo et al. 2013; Gilston et al. 2016). However, no structures are available for CP with Zn^2+^ ions bound in either site.

To better understand the role of CP in modulating staphylococcal biology and pathogenesis, we report here studies of Zn^2+^ binding by CP and its effect on *S. aureus* growth and biofilm formation. X‐ray crystal structures reveal how Zn^2+^ ions are bound in the two CP transition metal binding sites, confirming the role of the S100A9 C‐terminal tail in the His_6_ site. Site‐directed and truncation mutations explore the contributions of the tail to the binding of Zn and antimicrobial activity. Growth and biomass accumulation assays probe the contributions of the S100A9 C‐terminal tail to *S. aureus* growth restriction by CP. These results suggest that CP functions via multiple mechanisms, not all of which are dependent on transition metal sequestration.

## 2 RESULTS

### 2.1 Zn sequestration by CP inhibits *S. aureus* growth and biomass accrual

**FIGURE 1:**
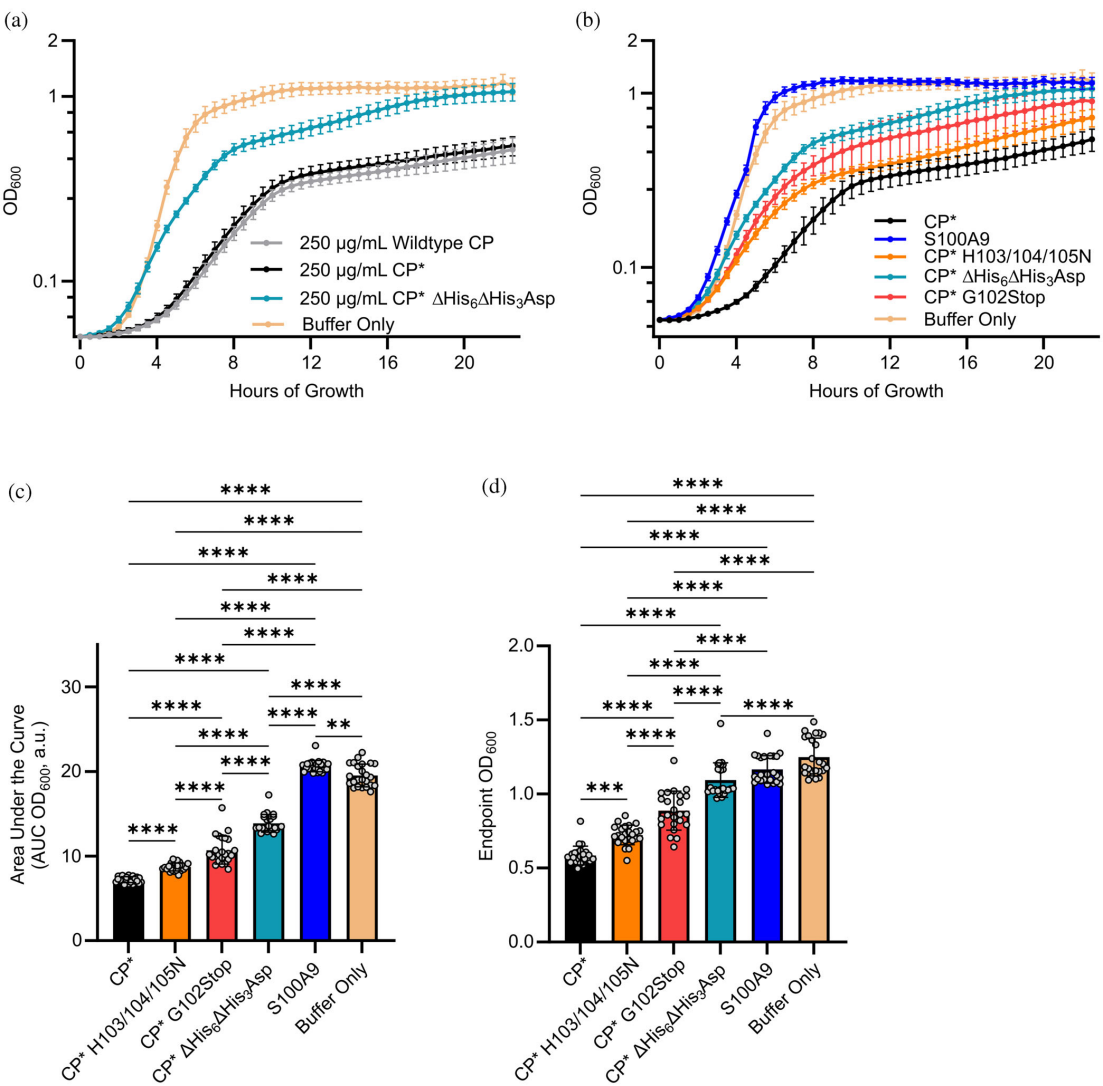
CP metal‐binding sites are critical for anti‐staphylococcal activity. (a) Growth of *S. aureus* in 1:1.5::TSB:Buffer conditions with CP, CP*, or CP* without transition metal binding (CP∆His_6_ ∆His_3_Asp). All cultures were monitored for changes in OD_600_ every 30 min of growth at 37°C, continuously shaking in a total of 150 μL of media. Data represent three biological and eight total replicates, normalized to starting OD_600_; mean and SD. (b) Growth of *S. aureus* in 1:1.5::TSB:Buffer conditions with and without CP* or variants at a concentration of 250 μg/mL. Data represent three biological and eight total replicates, normalized to starting OD_600_; mean and SD. CP* ∆His_6_∆His_3_Asp data is shared across (a) and (b), where baseline is re‐normalized to account for differences. (c) Statistical comparison of area under the curve (AUC) values via ordinary one‐way ANOVA with Tukey's multiple comparison test with a single pooled variance. (d) Statistical comparison of endpoint OD_600_ values via ordinary one‐way ANOVA tests with Tukey's multiple comparison test with a single pooled variance, **p < 0.01, ***p < 0.005, ****p < 0.001.

Trace transition metal sequestration by CP can have a detrimental impact on bacterial growth in vitro and in vivo, but can also serve as a reservoir for metals that enable pathogens to evade nutritional immunity (Murdoch and Skaar 2022). Growth assays were performed with cultures treated with buffer alone or 250 μg/mL of CP to validate the previously observed antimicrobial activity of calprotectin against *S. aureus*. CP treatment of nutrient‐rich media results in clear growth defects (Figure 1).

CP readily forms disulfide cross‐linked oligomers (Zackular et al. 2015). To minimize cross‐linking in biophysical experiments and functional assays, many studies incorporate the well‐characterized Cys‐Ser mutations of S100A8 (C42S) and S100A9 (C3S) (Hunter and Chazin 1998). This CP variant will be referred to as CP*. Assays comparing growth in the presence of CP* versus wild‐type CP revealed no significant differences (Figure 1a), validating that these mutations do not alter CP activity against *S. aureus*.

To directly test the hypothesis that this antimicrobial activity is attributable to sequestration of Zn^2+^, the growth assay was also performed with two previously reported CP* mutants: CP* H103/104/105N, which is able to bind Zn^2+^ and Cu^2+^ normally but is unable to bind Mn^2+^ or Fe^2+^ with high affinity, and CP* ∆His_6_∆His_3_Asp, which is unable to bind any transition metals with high affinity (Damo et al. 2013). At 8 and 24 h, the loss of both the His_6_ and His_3_Asp sites (CP* ∆His_6_∆His_3_Asp) resulted in only a limited repression of growth, whereas the CP* H103/104/105N mutant resulted in a substantial repression, although not to the level of CP* (Figure 1b–d). These data support previous studies showing that the role of transition metal binding is central to the antimicrobial activity of CP and that the sequestration of trace transition metals in the His_3_Asp and His_6_ sites is critical to the observed anti‐staphylococcal activity.

### 2.2 Structure of CP with a Zn^2+^ ion bound in the His_6_ site

Despite CP sequestration of zinc being important to inhibiting microbial growth, knowledge of the molecular mechanisms of Zn^2+^ binding at its two distinct transition metal binding sites is lacking. To define how CP chelates Zn^2+^ ions, we set out to determine the X‐ray crystal structure of Ca^2+^‐loaded, Zn^2+^‐bound CP*. Crystallization of Zn^2+^‐binding proteins is notoriously difficult, complicated by Zn^2+^‐dependent protein aggregation in solution (Loes et al. 2019). Crystallization of Zn^2+^‐loaded CP both by soaking Zn^2+^ ions into Ca^2+^‐loaded CP* crystals and co‐crystallizing in the presence of Zn^2+^ ions was undertaken. However, despite considerable effort, inclusion of more than 0.7 molar equivalents of Zn^2+^ in the buffer resulted in precipitation, even in the presence of MgCl_2_, which is known to improve S100 protein solubility. Dynamic light scattering (DLS) was used to examine the state of oligomerization of CP under different Zn^2+^ concentrations (Figure S1, Supporting Information). In the absence of Zn^2+^, CP* displays a monodispersed peak with decay times in the autocorrelation function indicative of its existence primarily in the tetrameric state. However, upon the introduction of Zn^2+^, a population of higher‐order oligomers indicated by higher decay times is observed along with increased polydispersity (Figure S1). This indicates that CP* undergoes Zn^2+^‐dependent oligomerization and aggregation, presumably reflecting changes in electrostatics at the surface upon binding to Zn^2+^ that promote oligomerization.

**FIGURE 2:**
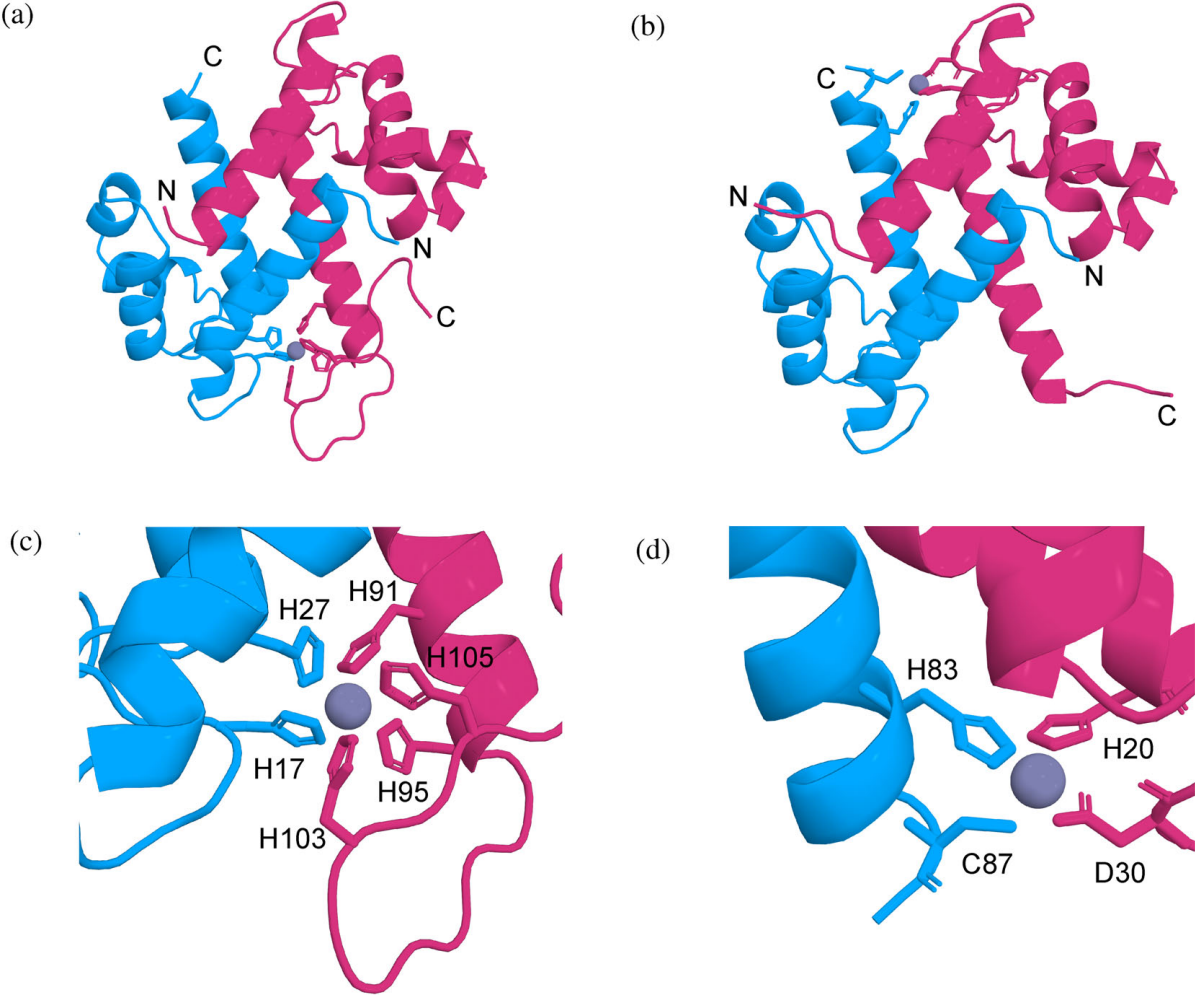
X‐ray crystal structures of the Zn^2+^‐bound Ca^2+^‐loaded CP dimer of CP* (PDB ID: (8SJC) and Ca^2+^‐loaded CP* H87C (8SJB). Ribbon diagrams in panels (a) and (b) depict the CP* and CP* H87C dimers with S100A8 colored blue and S100A9 magenta. Ca^2+^ ions are not shown here for clarity, while Zn^2+^ ions are depicted in gray. The complete tetramer with Ca^2+^ ions is shown in Figure S4. The close‐up view of the unique His_6_ site in panel (c) highlights the six histidine residues that chelate the Zn^2+^ ion. The close‐up view of the canonical His_3_Asp site of the H87C variant (d) shows the residues responsible for chelating the Zn^2+^ ion.

Crystallization of Zn^2+^‐bound CP* proved successful with sub‐stoichiometric levels of Zn^2+^ in solution, yielding crystals that diffracted to 1.87 Å. Refinement of the data produced the expected symmetric dimer of heterodimers with Ca^2+^ ions in the EF‐hands and Zn^2+^ ions bound in both His_6_ sites. The overall structure is very similar to those previously reported for Ca^2+^, Ca^2+^ + Mn^2+^, and Ca^2+^ + Ni^2+^ states, with pairwise C_α_ RMSDs of 0.18, 0.24 Å, and 0.27 Å, respectively (Figures S2 and S4a,b) (Damo et al. 2013; Nakashige et al. 2018). The Zn^2+^ occupancy was 0.51 and 0.54 in the two His_6_ sites of the tetramer, reflecting the substoichiometric levels of Zn^2+^ in the buffer (Table S2a). As expected, the Zn^2+^ ions are coordinated by the N^ε2^ atoms of His17 and His27 of S100A8 and His91, His95, His103, and His105 of S100A9 (Figures 2a,c and S3a,c). Details on the coordination geometry are provided in Tables S3a and S3b. Notably, binding of the Zn^2+^ ion in the His_6_ site results in structural stabilization of the otherwise flexible C‐terminal tail as well as an increase in the dimer interface.

### 2.3 Structure of CP with a Zn^2+^ ion bound in the canonical His_3_Asp site

Considering the limited solubility of CP in the presence of Zn^2+^ and the preferential binding in the His_6_ site observed for the WT protein, we reasoned that it might be possible to obtain a structure with a Zn^2+^ ion bound in the canonical His_3_Asp site by abrogating transition metal binding in the His_6_ site. Crystallization trials were therefore performed on a variety of variants prepared with reengineered CP* metal binding sites with Zn^2+^ added at 0.7 molar equivalents to Ca^2+^‐loaded CP* in the presence of 2 mM MgCl_2_ to prevent aggregation. Among these, a CP* variant carrying the H87C mutation in the His_3_Asp site, along with the ΔHis_6_ mutations, crystallized with a Zn^2+^ ion bound in the His_3_Asp site. The crystals diffracted to 1.74 Å, and refinement of the data produced the expected tetrameric structure. In this case, only one of the two His_3_Asp sites within the tetramer had a Zn^2+^ ion bound with significant occupancy (0.6) (Figures S4a,b and Table S2a). The overall structure was again similar to previously reported CP structures including that of CP with a Zn^2+^ bound in the His_6_ site (Figure S2). Ca^2+^ ions are bound in the EF‐hands, and the Zn^2+^ ion is coordinated by residues His83 (N^ε2^) and Cys87 (S^ϒ1^) of S100A8 and His20 (N^ε2^) and Asp30 (O^δ1^) of S100A9, thus forming an overall distorted tetrahedral geometry (Tables S4a and S4b and Figures 2b,d and S3b,d).

Remarkably, electron density for the C‐terminal tail is observed in this structure through to Gly100 (Figures 2b and S4c,d). In all previous CP structures, when an ion is bound in the His_6_ site, the C‐terminal tail of S100A9 wraps around the metal ion and well‐defined electron density is observed (Damo et al. 2013). In contrast, when no ion is bound in the His_6_ site, electron density is observed only through Glu92 (e.g., PDBID 1XK4). Hence, this is the first example in which density for the S100A9 C‐terminal tail is observed when it is not engaged in coordinating a transition metal. This conformation is stabilized by crystal contacts, as confirmed by analysis of the crystal packing environment.

### 2.4 High‐affinity binding of Zn^2+^ in the His_6_ site does not require His residues from the C‐terminal tail

**FIGURE 3:**
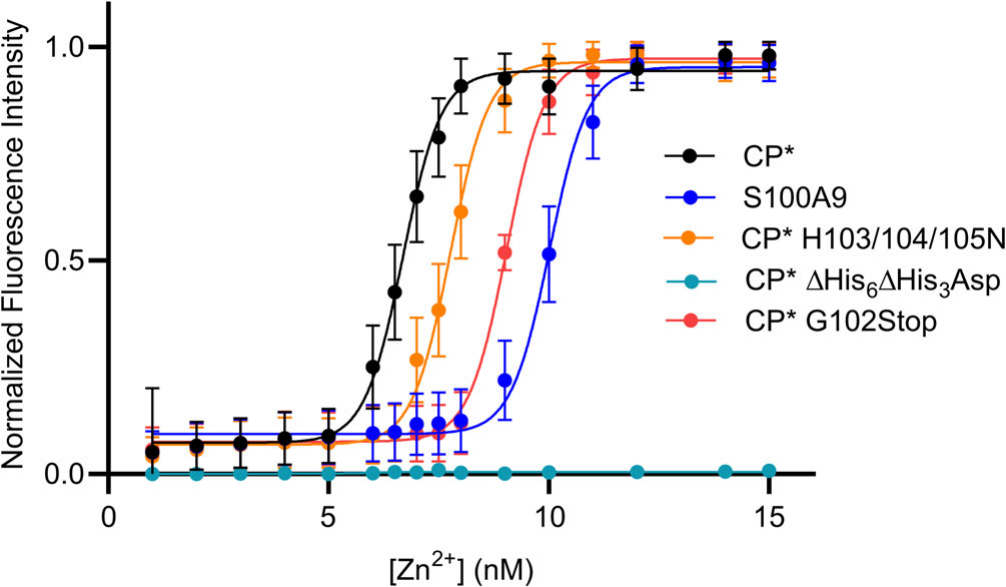
Mutation of S100A9 C‐terminal tail has limited effect on CP Zn^2+^ affinity. Determination of zinc binding affinity of CP and variants by a competitive chelator approach. Each binding curve was fit individually, and then the reported apparent *K*_d_ was calculated by averaging the individual values obtained for triplicate independent measurements. The Zn affinity of CP* = 0.16 ± 0.07 pM, CP H103/104/105N = 0.21 ± 0.03 pM, CP G102Stop = 0.22 ± 0.07 pM, S100A9 = 0.48 ± 0.04 pM, CP ∆His6∆His3Asp = n/a.

The observation that all six histidine residues chelate the Zn^2+^ ion in the His_6_ site appears inconsistent with earlier works, which showed that mutation of His103 and His105 in the C‐terminal tail did not significantly alter overall Zn^2+^ binding affinity. To resolve this apparent discrepancy, we turned to the competitive chelator approach applied previously by Nolan and coworkers to measure Zn^2+^ binding parameters for CP with higher accuracy (Cunden et al. 2017). Two CP* variants were used for this study: H103/104/105N, which mutates the His103 and His105 side chains, and G102Stop, which deletes the entire C‐terminal tail. Fitting of the concentration‐dependent increase in fluorescence signal to a standard binding curve provided apparent *K*_d_ (*K*_dAPP_) values for the average of the two Zn^2+^ sites: CP* (0.16 ± 0.07 pM), CP* H103/104/105N (0.21 ± 0.03 pM), CP* G102Stop (*K*_d_ 0.22 ± 0.07 pM), negative control CP* ∆His_6_∆His_3_Asp (n/a) (Figure 3). These results suggest that high‐affinity Zn^2+^ binding is retained even in the absence of the C‐terminal tail. However, as the chelator assay measures average affinity across both binding sites, it does not distinguish individual contributions. This leaves open the question of how much the three C‐terminal histidine residues (His103, His104, His105) contribute to site‐specific Zn^2+^ binding, especially given the structural evidence for preferential occupancy at the His_6_ site.

### 2.5 Modification to the C‐terminal tail of S100A9 lessens its antimicrobial effects on *S. aureus*

Additional growth assays were performed to determine if the C‐terminal tail has a specific role in the antimicrobial activity of CP. For this analysis, *S. aureus* growth was compared in the presence of 250 μg/mL CP* versus the corresponding amounts of CP* ∆His_6_∆His_3_Asp, CP* G102Stop, CP* H103/104/105N, or S100A9 (control) (Figure 1b–d). As expected, S100A9 alone had a minimal impact on *S. aureus* growth compared to buffer alone, whereas CP* treatment of the media results in clear growth and carrying capacity defects (Figure 1b–d). The loss of both transition metal‐binding sites (∆His_6_∆His_3_Asp) results in a significant reversion of the growth defects observed with CP* treatment, whereas both H103/104/105N and G102Stop variants result in intermediate phenotypes. This suggests that the S100A9 C‐terminal tail and its histidine residues significantly impact *S. aureus* growth.

### 2.6 *Staphylococcus aureus* biomass accumulation is differentially affected by metal sequestration of CP variants

**FIGURE 4:**
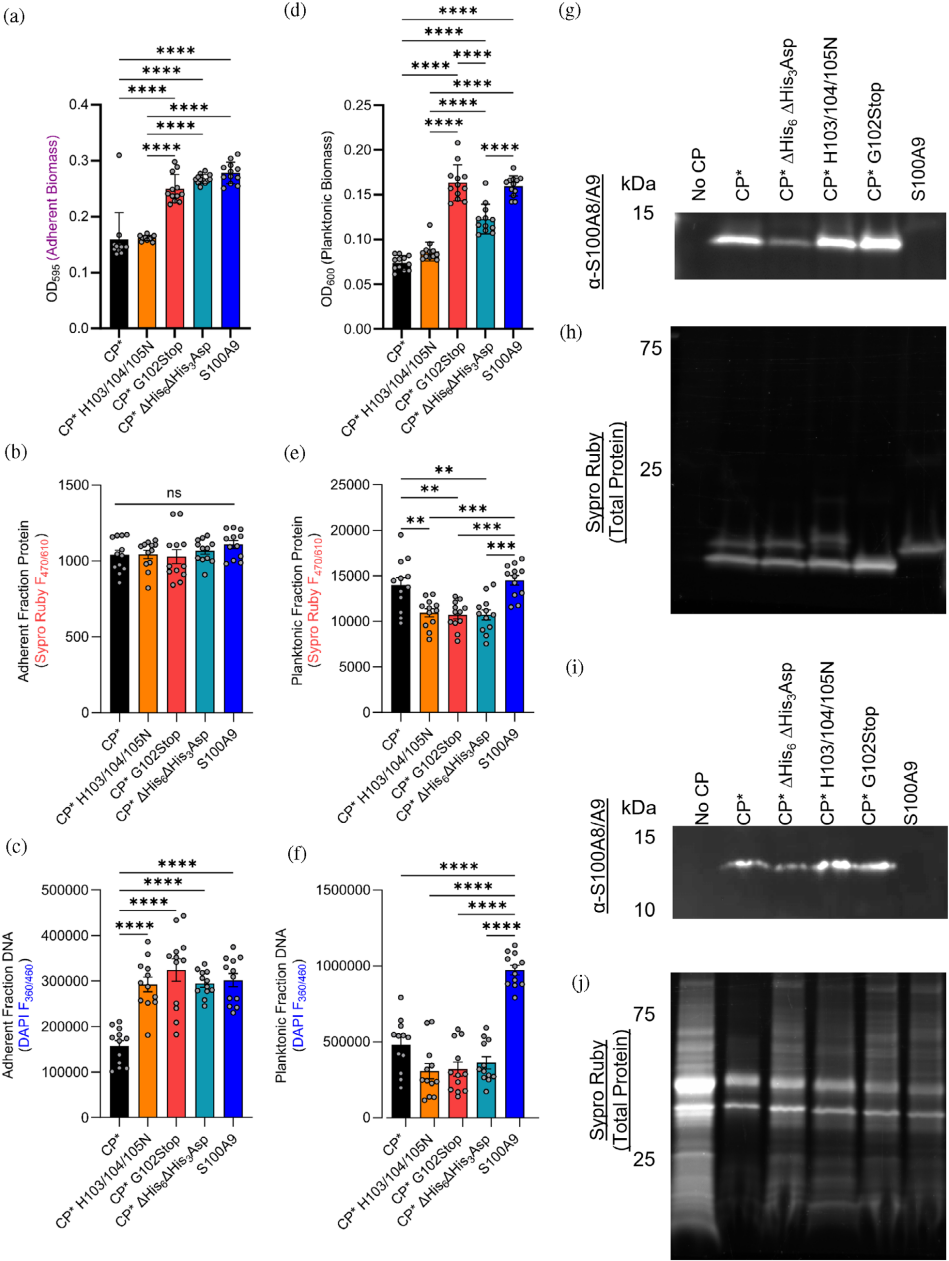
Early *S. aureus* biomass accumulation significantly differs in the absence of the S100A9 C‐terminal tail. Measurement of adherent (a–c) and planktonic (d–f) *S. aureus* biomass grown under 1:1.5 TSB:Buffer conditions with and without CP* or variants for 12 h. Cultures were either stained with crystal violet for total biomass (a, d), SYPRO Ruby for total protein (b, e), or DAPI for total DNA (c, f). Two biological and 12 total technical replicates were used for each condition. (g) Representative western blot and (h) SYPRO Ruby stained SDS PAGE gel depicting the abundance of S100 proteins in media pre‐inoculation. (i) Representative western blot and (j) SYPRO Ruby stained gel of S100 protein variants treated cultures 12 h after inoculation. Panels (i) and (j) are representative of at least triplicate results (see Figure S5). All statistical comparisons made by ordinary one‐way ANOVA tests with Tukey's multiple comparison test with a single pooled variance, **p < 0.01, ***p < 0.005, ****p < 0.001.

Simple liquid growth assays are insufficient in this case to definitively characterize defects in *S. aureus* growth and pathogenesis associated with the S100A9 C‐terminal tail. Since adherent biomass and biofilm formation are clinically relevant features that determine outcomes of infection with *S. aureus* (Schilcher and Horswill 2020; Vestby et al. 2020), the fractions of bacterial communities (adherent and nonadherent/planktonic) were quantified using crystal violet (CV) assays, in addition to parallel cultures exposed to SYPRO Ruby and to DAPI (Atkin et al. 2014; Flemming and Wingender 2010; Stoodley et al. 2002). The cyclical nature of biomass accrual and dispersal has been described biochemically, morphologically, and clinically (Atkin et al. 2014; Cruz et al. 2018; Flemming and Wingender 2010; Haque et al. 2021; Kaplan et al. 2012; Macia et al. 2014; Schilcher and Horswill 2020; Stoodley et al. 2002). CP has known roles in altering *S. aureus* biofilm formation, which are reflected in Figure 4a,b (Enriquez et al. 2023; Wakeman et al. 2016). At 12 h of still culture, differences in adherent (stained by crystal violet, OD_595_; Figure 4a) and planktonic (soluble, OD_600_; Figure 4d) biomass are observed, associated with the trace transition metal binding activity of CP. In parallel to Figure 1b, treatment of *S. aureus* cultures with CP* results in significant decreases in both planktonic (Figure 4d) and adherent (Figure 4a) biomass as compared to the negative control of S100A9. In cultures treated with CP* H103/104/105N, which maintains its ability to bind Zn^2+^ and Cu^2+^, but not Mn^2+^ nor Fe^2+^, significant decreases in biomass are observed similar to CP*‐treated cultures. In the case of treatment with ∆His_6_∆His_3_Asp, decreased planktonic but not adherent biomass is observed (Figure 4d), whereas treatment with G102Stop results in no decrease in either adherent or planktonic biomass accrual. These data indicate that sequestration of trace transition metals by CP alters the accumulation of planktonic and adherent *S. aureus* biomass, but that there is a distinct contribution from the S100A9 C‐terminal tail (Atkin et al. 2014; Cruz et al. 2018; Flemming and Wingender 2010; Haque et al. 2021; Kaplan et al. 2012; Macia et al. 2014; Schilcher and Horswill 2020; Stoodley et al. 2002).

Planktonic and adherent biomass are composed of complex molecules, including intra‐ and extra‐cellular DNA, protein, and polysaccharides (Atkin et al. 2014; Cegelski 2015; Donlan 2001; Idrees et al. 2021; Sauer et al. 2022). To gain insight into the composition of biomass accrued in response to treatment, parallel cultures of *S. aureus* exposed to CP* and the other variants were separated into planktonic and adherent fractions and stained with SYPRO Ruby and DAPI. In the adherent fraction (Figure 4a–c), the treatments resulted in a similar concentration of total intra‐ and extra‐cellular protein as quantified by SYPRO Ruby staining. However, significantly decreased DAPI staining indicating a statistically significant decrease in DNA abundance was observed in CP*‐treated cultures compared to all other conditions. In contrast, although the CV staining for treatment with H103/104/105N showed the fractions of adherent and planktonic biomass were similar to CP*, the amounts of SYPRO Ruby and DAPI staining were significantly higher, similar to S100A9‐treated cultures (Figure 4a–c). Crystal violet is known to bind all major components of bacterial biomass (protein, DNA, lipids, and polysaccharides) regardless of cell viability. Therefore, the differences observed for H103/104/105N treatment are likely not reflecting differences in DNA and total protein but rather may represent differences in extracellular matrix in the form of polysaccharides.

In the planktonic fractions (Figure 4d–f), CP*‐treated cultures exhibit low biomass (OD_600_), but high SYPRO Ruby staining and a somewhat higher DNA content by DAPI staining compared to the other variants. Since the differences captured by total biomass are incompletely explained through quantification of total protein and total DNA, these experiments suggest that the observed differences in biomass arise from differences in polysaccharide and/or viable cellular content.

### 2.7 CP* interaction with *S. aureus* is influenced by the His_3_Asp, His_6_, and the S100A9 C‐terminal tail

The observed antimicrobial mechanism of action associated with the S100A9 tail may result from it directly interacting with *S. aureus*. To test this hypothesis, differences in CP and total protein content were measured 12 h after inoculation. As expected, pre‐inoculation measurements with 250 μg/mL of protein added to the media show there is little difference in protein content in the different samples (Figure 4g,h). Multiclonal anti‐S100A8/A9 antibodies successfully detect all CP proteins used in these experiments (Figure 4g). The observation of a single band for the G102Stop variant arises because the truncation of the S100A9 C‐terminal tail results in the S100A9 subunit having a very similar mass to S100A8, which causes the bands to overlap. The lower level of ∆His_6_∆His_3_Asp staining compared to other variants in Figure 4g is puzzling at first glance. However, staining with SYPRO Ruby in Figure 4h indicates the amount of this protein is about the same, which implies that the ensemble of mutations in this variant alters the interaction with the antibody leading to weaker staining in the blot (Figure S5).

The protein associated with bacterial biomass was measured 12 h after inoculation with *S. aureus* (Figure 4i,j). The amount loaded into each well was standardized so that staining reflected differences in protein abundance across conditions. This normalization provides an additional measure of bacterial abundance by using comparison of nonspecific dye/antibody capture by *S. aureus* surface proteins at ~50 and ~37 kDa as reporters of biomass. *Staphylococcus aureus* produces multiple highly abundant proteins such as SpA that nonspecifically capture antibodies as a strategy to evade the immune system. SpA binds to the Fc region of immunoglobulins and therefore “nonspecific” bands are common on immunoblots against *S. aureus* lysates (Cheung et al. 1997). Comparing different treatments shows the expected trend with the most *S. aureus*‐associated proteins in buffer‐only controls, the least with CP* treatment, and the other variants in between, while immunoblotting intensities are relatively similar (Figures 4i,j and S5). The observation that total protein content is substantially lower only for CP* shows that both metal binding (∆His_3_Asp∆His_6_) and residues in the S100A9 C‐terminal tail (H103/104/105N, G102Stop) are required for interaction with *S. aureus*.

## 3 DISCUSSION

The loss of CP is detrimental for the host in combatting infection with numerous pathogens, including *S. aureus* (Edgeworth et al. 1991; Rammes et al. 1997; Tardif et al. 2015). Previous studies of the antimicrobial mechanisms of CP against *S. aureus* revealed that the binding of Zn^2+^ and Mn^2+^ is essential to its antimicrobial activity, while noting other mechanisms contribute (Brophy and Nolan 2015; Damo et al. 2013; Rosen and Nolan 2020; Sohnle et al. 2000; Zygiel and Nolan 2018). This study focused on obtaining a clearer understanding of the Zn^2+^‐mediated antimicrobial activity of CP against *S. aureus*. Determining structures of CP* with bound Zn^2+^ ions posed a substantial challenge due to the low solubility of CP* upon addition of Zn^2+^, which required using substoichiometric levels of Zn^2+^ in the crystallization buffers. Our structures provided direct confirmation of how Zn^2+^ ions are coordinated in CP's His_3_Asp site and showed that all six residues are engaged in the His_6_ site. Interestingly, high‐affinity Zn^2+^ binding was retained even after mutation of the His_6_ site Zn^2+^ ligands His103 and His105 or removal of the entire S100A9 C‐terminal extension (tail). However, detailed investigation of growth in the presence of CP* variants showed differences in the growth of the adherent versus nonadherent fractions of *S. aureus*, suggesting a contribution to CP* activity that is independent of sequestration of zinc.

We have previously shown that the S100A9 C‐terminal tail is essential for Mn^2+^ binding and the antimicrobial activity of CP*, but is not required for binding of Zn^2+^ (Damo et al. 2013). Three‐dimensional structures of CP* have been determined with Ca^2+^ (PDB‐1XK4), Ca^2+^+Mn^2+^ (PDB‐4GGF), and Ca^2+^+Ni^2+^ (PDB‐6DS2), but none with Zn^2+^ ions bound until those reported here. The structures we determined are similar in overall architecture to those previously reported. However, it is surprising that in our structure of CP* ∆His_6_H87C with Zn^2+^ bound in the canonical His_3_Asp site, we observe clear electron density for the S100A9 C‐terminal tail. The tail is known to be flexible in solution and disordered in all previous CP structures obtained with no metal ion bound in the His6 site. However, careful inspection of crystal packing led to the discovery of unique crystal contacts that are presumably stabilizing the C‐terminal tail in this crystal form.

The structure of CP* with a Zn^2+^ ion bound revealed hexacoordinate Zn^2+^ binding in the His_6_ site as is observed for Mn^2+^ and Ni^2+^. However, we confirm that only four of the six His residues in the His_6_ site are required for high‐affinity Zn^2+^ binding in vitro (Figure 2). The Zn^2+^ binding curves obtained in solution show that the two transition metal binding sites in CP are indistinguishable. So why then is the His_6_ site occupied but not the His_3_Asp site in the structure of CP* obtained with substoichiometric levels of Zn^2+^? We attribute this observation to preferential crystallization of one species versus the other, that is, that the two half Zn^2+^‐loaded states are present in equilibrium in solution but that crystallization occurs much more readily for the species with a Zn^2+^ ion in the His_6_ site as opposed to the His_3_Asp site.

Our functional assays were consistent with past reports that metals are central to *S. aureus* growth (Cassat and Skaar 2012; Cho et al. 2015; Kehl‐Fie et al. 2011; Nakatani et al. 2005; Wakeman et al. 2016). While CP* heavily restricts *S. aureus* growth across 24 h of culture, the variant with mutation of His103, His104, and His105 has a lesser effect, and knockout of both the His_3_Asp and His_6_ sites results in only a minor reduction in *S. aureus* growth. Measurement of adherent and nonadherent *S. aureus* culture fractions suggested that *S. aureus* biomass accrual is impacted most by treatment with CP* versus the His103/104/105 variant and the variant with the S100A9 tail truncated (G102Stop). In particular, biomass accrual studies using G102Stop‐ or H103/104/105N‐treated cultures exhibit different patterns of planktonic and adherent biomass. For example, treatment with the G102Stop mutant resulted in increased biomass compared to even the ∆His_6_∆His_3_Asp mutant (Figure 4a–f). These differences are present across measurements of total biomass in Figure 4a,d, and consistent with analyses of total protein (Figure 4b,e) and total DNA (Figure 4c,f). From these analyses, adherent biomass is associated with ~10% of total biomass at these short timepoints, consistent with models of the biofilm lifecycle (Enriquez et al. 2023; Rumbaugh and Sauer 2020; Sauer et al. 2022). Loss of the S100A9 tail is also associated with decreased binding of *S. aureus* similar to the loss of both His_3_Asp and His_6_ transition metal binding sites (Figure 4g‐j). Thus, while some of the effect of CP* is reduced by mutations of His103, His104, and His105, the remaining residues in the CP* tail have a clear effect on *S. aureus* biology and pathogenesis (Kerkhoff et al. 1999; Rammes et al. 1997; Siegenthaler et al. 1997; Sohnle et al. 2000).

The distinct phenotype observed upon deletion of the S100A9 C‐terminal tail requires further study of the biochemical and microbiological mechanisms of CP in mediating *S. aureus* pathology. Collectively, our results reveal the importance of the S100A9 C‐terminal tail in the binding of Zn^2+^ by CP at the host–microbe interface and suggest additional roles in biofilm formation and biomass accrual for the tail independent of its metal‐binding activity, potentially associated with the interaction between this protein region and *S. aureus*.

## 4 MATERIALS AND METHODS

### 4.1 Media and reagents

Tryptic soy (TSB) was obtained from BD, where phosphate buffered saline (PBS) was obtained from Gibco. Ninety‐six well, sterile, tissue‐culture treated, clear polystyrene flat‐bottom plates (DNAse‐, RNAse‐, DNA‐, and pyrogen‐free) were obtained from CytoOne. Sigma‐Aldrich sourced zinc chloride (>98% purity), manganese (II) chloride tetrahydrate (ReagentPlus, 99% purity), anhydrous calcium chloride (granular, <7.0 mm, >93% purity), and iron (III) chloride (97% purity). N, N, N′, N′‐Tetrakis‐(2‐pyridyl‐methyl)‐ethylene‐diamine (TPEN) and Chelex 100, molecular grade, 200–400 mesh were obtained from BioRad. Molecular biology grade DMSO was obtained from MilliporeSigma, and fluorescent probe Zinpyr‐4 (ZP4) was obtained from Santa Cruz Biotechnology. EPOCH 2 Microplate Spectrophotometer was obtained from Biotek.

### 4.2 Expression and purification of CP and CP variants

Calprotectin (CP) and all variants were prepared as previously reported (Damo et al. 2013; Kehl‐Fie et al. 2011; Maurakis et al. 2019). To facilitate structural and biophysical studies, all constructs contained Ser substitutions for the native Cys residues (S100A8 C42S, S100A9 C3S) (Damo et al. 2013). Figure 1a confirms that this variant, termed CP*, has comparable antimicrobial activity to CP. The mutations made in each subunit are listed in Table 1 along with the abbreviation used for each variant.

**TABLE 1:** S100A8 and S100A9 mutations in CP variants.

Variant	S100A8 mutations	S100A9 mutations	ε (extinction coefficient)	MW (kDa)
CP*	Cys42Ser	Cys3Ser	18,450	23.95
H103/104/105N	–	His103, His104, His105N	18,450	23.88
∆His_6_	His17, His27	His91, His95, His103, His105	18,450	23.85
∆His_3_Asp	His83, His87	His20, Asp30	18,450	23.85
H87C ΔHis_6_	His87Cys, His17, His27	His91, His95, His103, His105	18,450	23.82
∆His_6_∆His_3_Asp	His17, His27, His83, His87	His20, Asp30, His91, His95, His103, His105	18,450	23.76
G102Stop	–	Truncation at Gly102	18,450	22.83
S100A9	N/A	Cys3Ser	6990	13.11

### 4.3 Bacterial strains and stock production

All microbiological experiments use the *S. aureus* bacterial strain Newman. All bacterial stocks were maintained at −80°C in 20% glycerol. Prior to each experiment, bacteria were streaked onto tryptic soy agar (TSA; 2% agar) and grown at 37°C overnight. From these cultures, single colonies were transferred to 5 mL of tryptic soy broth. Liquid cultures were then grown to stationary phase at a 45° angle, 180 rpm, and 37°C for experimental use.

### 4.4 Growth curves

From bacterial overnights grown in nutrient‐rich media, cultures were spun down at 4000*g* for 5 min, washed with sterile PBS, and normalized to an absorbance of OD_600_ = 1.0 ± 0.05. In a ratio of 10 μL/5 mL (1:2000), normalized culture was inoculated into growth media, of which 90 μL was aliquoted into each well of a 96‐well plate (CytoOne). Growth media were defined as in previous studies as a 1.5:1 ratio (total 150 μL of nutrient rich media to buffer, with or without exogenous protein) (Damo et al. 2013; Kehl‐Fie et al. 2011; Wakeman et al. 2016). Buffer for all growth assays was composed of 20 mM Tris Base, 100 mM NaCl, 3 mM CaCl_2_, and 5 mM β‐mercaptoethanol in distilled water. All protein samples had concentrations determined via UV–Vis spectroscopy and extinction coefficients, as listed in Table 1. Unless otherwise noted, all proteins were applied to cultures at a final concentration of 250 μg/mL. Measurements were made across at least triplicate biological and triplicate technical replicates using an EPOCH 2 microplate reader (Biotek). Cultures were kept at 37°C and shaken in a continuous orbital pattern over 24 h, where OD_600_ measurements (with 96‐well plate lid on) were taken at 30 min intervals.

### 4.5 CV, SYPRO Ruby, and DAPI assays

CV, SYPRO Ruby, and DAPI assays and associated statistical work were performed using GraphPad Prism 10, R, and Stata v17 software, using procedures detailed in Windows GPvf (2024), O'Toole (2011), Team RC (2018), and StataCorp (2021). One‐hundred and seventy microliters of SYPRO Ruby Film Tracer Dye (ThermoFisher) was applied undiluted to wells after the separation of planktonic and adherent biomass fractions, whereas DAPI (ThermoFisher) was used at a 1:500 dilution in Ultrapure water. After each staining protocol, per manufacturer's instruction, samples were spun down and washed before being measured via Cytation 5 plate reader. Media conditions and preparation for growth curves and CV assays were identical; however, data were collected from independent samples. Measurements were collected from unshaken cultures to permit increased bacterial aggregation and adherence and for consistency with published literature. Measurements were made across quadruplicate biological and triplicate technical replicates.

### 4.6 Immunoblotting

Overnight cultures of strains of interest were diluted 1:100 v/v in 3 mL (1.5:1S100 protein with Buffer:TSB) fresh media with or without stress, as listed per lane and per figure. After back‐diluted cultures had grown at 37°C statically for 12 h, cultures were centrifuged and media were discarded. Triplicate pellets were resuspended in 400 μL sterile PBS containing 10 mM MgCl_2_ and subjected to treatment with lysostaphin for 1 h at 37°C. After treatment, samples were treated with 1% IGEPAL in PBS and 250 μM PMSF. Once incubated on ice for 10 min, samples were transferred to 2 mL Lysing Matrix B tubes and subjected to three rounds of bead beating at a speed of 5.0 for 45 s, with incubation on ice between cycles. Samples were then treated with 20 μg of DNAse I, cell debris was pelleted, and samples normalized by protein concentration were then run on two parallel gels for 35 min at 200 V. Per replicate, one gel was subjected to SYPRO Ruby staining, following the extended protocol provided by ThermoFisher (Catalog ID: S12000). The other gel was transferred to a nitrocellulose membrane for 18 min at 25 V and 1.0 A in a semi‐wet BioRad system and blocked using TBST w/5% w/v EasyBlocker (GeneTex Catalog No: GTX425858) for >1 h rocking at room temperature. The primary S100A8/A9 antibody (Abcam: ab288715) was applied at 1:1000 dilution at 4°C rocking overnight, washed with TBST three times, and treated with secondary antibody (Licor Donkey anti‐Rabbit IRDye® 680RD) for 1 h rocking at room temperature. Blots were then imaged in colorimetric and fluorescent channels on a BioRad ChemiDoc imaging system.

### 4.7 Crystallization of Ca^2+^/Zn^2+^ CP and H87C (ΔHis_6_ ) mutant

Due to aggregation propensity of CP* in the presence of Zn^2+^ all crystallization trials were performed with substoichiometric level of Zn^2+^ (Loes et al. 2019). CaCl_2_ (2 mM) was added to CP before crystalizing in buffer containing 20 mM Tris at pH 7.5, 100 mM NaCl, 2 mM MgCl_2_, and 0.7 equivalents of Zn^2+^. The protein was concentrated to 15 mg/mL protein, after adding 0.7 eq. of ZnCl_2_ in a stepwise manner. CP* crystals appeared in VDX 24‐well crystallization trays (Hampton Research) using the hanging drop vapor diffusion method. This involved mixing 1 μL of protein solution with 1 μL of reservoir solution, and the crystals appeared after 5 days at 20°C in 1.0M sodium citrate (pH 5.2), 28% MPD.

A similar approach was used to crystallize the CP* ΔHis_6_ mutant. The protein was suspended in a buffer consisting of 20 mM HEPES at pH 7.5, 100 mM NaCl, and 2 mM CaCl_2_. Crystallization of the protein at a concentration of 10 mg/mL was achieved using the hanging drop vapor diffusion method. After 3 days at 20°C, crystals appeared in 0.1M HEPES pH 8.0, 0.05M ammonium acetate, and 25% PEG 3350. The crystals were soaked in a cryoprotective solution corresponding to the reservoir of their crystallization drop with 25% glycerol, followed by vitrification in liquid nitrogen.

### 4.8 X‐ray crystallography

X‐ray diffraction data were collected at a wavelength of 0.97857 Å, using the Advanced Photon Source beamline 21‐ID‐G at Argonne National Laboratory. The data were processed with hkl2000 (Morin and Sliz 2013; Otwinowski and Minor 1997). CP* crystallized in the P6_1_ space group and crystals diffracted to a maximal resolution of 1.87 Å. CP* ΔHis_6_ crystallized in the P2_1_2_1_2_1_ space group and crystals diffracted to a maximum resolution of 1.7 Å. Phasing of the data was performed using PHASER from the PHENIX package using the crystal structure of Mn^2+^ bound calprotectin PDBID: 4GGF as a search model (Adams et al. 2010). Refinement of the initial model was carried out by alternating cycles of manual rebuilding in COOT and cycles of refinement with PHENIX.REFINE (Adams et al. 2010; Emsley et al. 2010). Data collection and refinement statistics are provided in Table S1. Atomic coordinates and structure factors have been deposited in the Protein Data Bank under the accession number 8SJC for CP* and 8SJB for CP* ΔHis_6_. The quality of all structures was checked with MOLPROBITY. Structural biology figures were generated using PYMOL.

### 4.9 Preparation of metal free buffer and protein

Buffers and proteins were treated with Chelex 100, molecular grade, 200–400 mesh prior to all experiments requiring addition of Ca^2+^, Zn^2+^ or both to ensure that the starting materials were metal‐free. Prior to use, all resins were washed using 300 volumes of MilliQ water to remove salts and any contaminants. The wet resin was then added to the buffer or the protein using plastic utensils to avoid any metal contamination. The mixture was incubated overnight with slight agitation. The next day, buffers or proteins were separated from the resin by centrifugation and used immediately.

CP* proteins were denatured using 8M guanidinium chloride to disrupt all metal coordination at the metal binding sites. Subsequently, the protein was dialyzed against a metal‐free buffer containing 20 mM Tris (pH 8) and 150 mM NaCl to remove metal ions and facilitate protein refolding. Dynamic light scattering (DLS) analysis confirmed folding and oligomerization of the proteins post‐dialysis (data not shown).

### 4.10 Measurement of Zn affinity

A competition chelator method using the fluorescent probe Zinpyr‐4 (ZP4) was used to measure the affinity of the CP* proteins for Zn^2+^ (Cunden et al. 2017). Since the method measures affinity indirectly by competition, we report apparent dissociation constants (*K*_dAPP_). ZP4 was dissolved in DMSO to prepare a stock solution at 1.5 mM; then it was aliquoted and kept in the dark at −20°C. The buffer for CP* was 20 mM Tris at pH 8 and 150 mM NaCl +/− 2 mM Ca^2+^. Both protein and buffer were pre‐incubated with Chelex resin.

Prior to ZP4 addition, CP* was completely denatured using 6M guanidinium chloride to remove any high‐affinity metals bound to CP* during protein production, and it was subsequently refolded using the method described by Damo et al. (2013). To limit aggregation of CP*, a modification to the conventional protocol for this assay was implemented in which separate samples of CP* and ZP4 were made, each with a different Zn^2+^ concentration, as opposed to making incremental additions of Zn^2+^ into a mixture of CP* and ZP4. The protein was first mixed with ZP4. After 20 min of equilibration, the protein/ZP4 mixture was aliquoted into black Eppendorf tubes and ZnSO_4_ was added to bring the solution to the desired Zn^2+^ concentration. The mixture was allowed to equilibrate for 60 min prior to measurement. Mixtures and additions were adjusted to ensure each solution ended with the same dilution factor and a final concentration of 5 μM protein and 2 μM ZP4. The emission spectrum was recorded using a Horiba Jobin Yvon Fluoromax‐3 fluorimeter and a submicro fluorometer Starna quartz cuvette. The emission spectrum was recorded from 510 to 530 nm, using 490 nm as the excitation wavelength. All experiments were performed at least in triplicate.

The data were normalized (*F* − *F*_min_/(*F*_max_ − *F*_min_)) and fit by nonlinear least squares regression in GraphPad Prism to a standard 1:1 binding equation for both ZP4 and CP*. In this model, the two Zn^2+^‐binding sites in CP* are treated as equivalent (Windows GPvf 2024).

### 4.11 Dynamic light scattering

Data were collected for CP* in the absence and presence of Zn^2+^ at 25°C using a DynaPro NanoStar (Wyatt Technology, USA) with a 5‐s acquisition time and 10 acquisitions per measurement. The protein samples were prepared in 20 mM Tris at pH 8 and 100 mM NaCl at 10 mg/mL, with 0.0, 0.1, 0.2, 0.5, 0.7, and 1.0 eq. of ZnCl_2_. Both protein and ZnCl_2_ solutions were filtered using a 0.22 μm filter prior to titration. Ten independent readings were recorded for measuring the average hydrodynamic radius (*R*_*h*_).

### 4.12 Sequence alignment

Sequence alignments of S100A8 and S100A9 to other known S100 proteins were performed using Clustal Omega (Sievers et al. 2011).

## AUTHOR CONTRIBUTIONS

**Yasiru R. Perera:** Investigation; writing – original draft; conceptualization; methodology; validation; visualization; writing – review and editing; formal analysis. **Kyle T. Enriquez:** Conceptualization; investigation; writing – original draft; methodology; validation; visualization; writing – review and editing; formal analysis. **Aslin Rodriguez:** Methodology; validation. **Velia Garcia:** Methodology; validation. **Tae Akizuki:** Methodology; validation. **Anais Naretto:** Methodology; validation. **Melumo Togashi:** Methodology; validation. **Ryan Guillen:** Methodology. **Eric P. Skaar:** Conceptualization; supervision; project administration; funding acquisition; writing – original draft; writing – review and editing. **Walter J. Chazin:** Conceptualization; funding acquisition; writing – original draft; writing – review and editing; software; data curation; resources.

## Supporting information


**Figure S1.** Supporting Information.

## Data Availability

The data that support the findings of this study are available from the corresponding author upon reasonable request.
