# Supplementary material for: The C‐terminal extension of calprotectin mediates zinc chelation and modulates Staphylococcus aureus biomass accumulation
Source: Protein Sci. 2025 Sep 13;34(10):e70294. doi: 10.1002/pro.70294 (PMC12432413; doi:10.1002/pro.70294)
Supplement: Supplementary file 1 — Figure S1. Supporting Information. [file PRO-34-e70294-s001.docx]

**Table S1. X-ray crystallography data collection and refinement statistics**


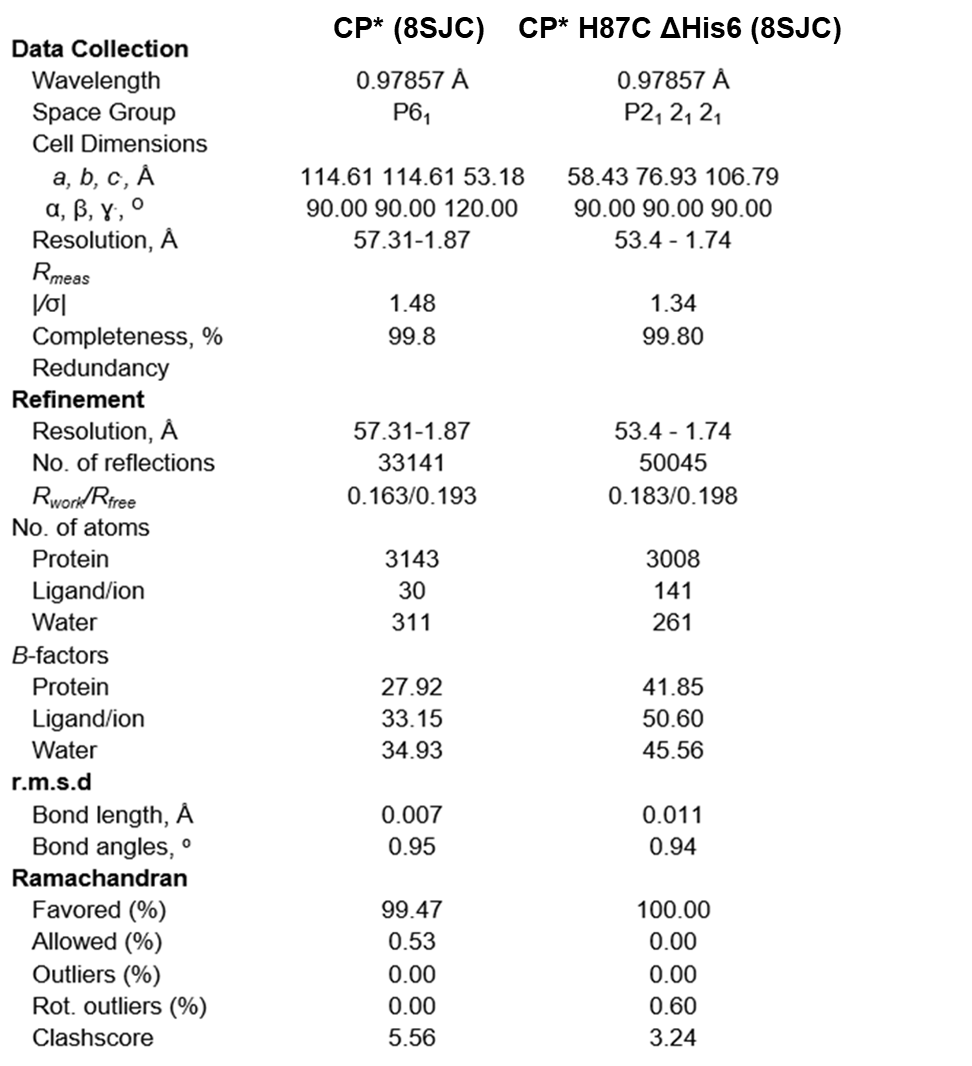


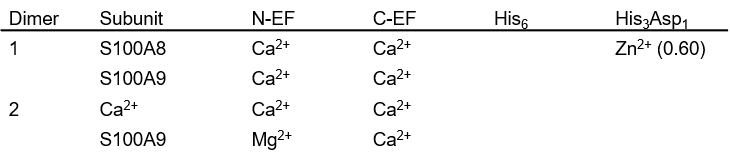

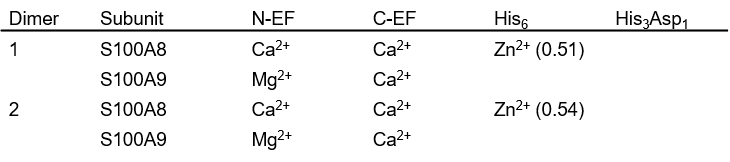


**Table S2B: The metal occupancy of Ca^2+^-loaded CP* H87C His3Asp site.**

**Table S2A: The metal occupancy of Ca^2+^-loaded CP* His_6_ site.**

**Table S3A: The bond distance for Zn^2+^-N^ε2^ of Ca^2+^-loaded CP* His_6_ site.**

|  |  | Bond Distance (A^o^) |
| --- | --- | --- |
|  | Residue | Zn^2+^ |
| S100A8 | Zn-N^ε2^_H17_ | 2.2 |
|  | Zn-N^ε2^_H27_ | 2.2 |
| S100A9 | Zn-N^ε2^_H91_ | 2.3 |
|  | Zn-N^ε2^_H95_ | 2.3 |
|  | Zn-N^ε2^_H103_ | 2.3 |
|  | Zn-N^ε2^_H105_ | 2.2 |

**Table S3B: The bond angle for N^ε2^_x_-Zn-N^ε2^_y_ of Ca^2+^-loaded CP* His_6_ site.**

|  | Bond Angle (^o^) |
| --- | --- |
| Residue | Zn^2+^ |
| N^ε2^_H91_-Zn-N^ε2^_H95_ | 90.4 |
| N^ε2^_H91_-Zn-N^ε2^_H105_ | 94.4 |
| N^ε2^_H91_-Zn-N^ε2^_H17_ | 88.1 |
| N^ε2^_H91_-Zn-N^ε2^_H27_ | 85.4 |
|  |  |
| N^ε2^_95_-Zn-N^ε2^_H105_ | 92.1 |
| N^ε2^_H105_-Zn-N^ε2^_H17_ | 80.9 |
| N^ε2^_H17_-Zn-N^ε2^_H27_ | 88.9 |
| N^ε2^_H27_-Zn-N^ε2^_H95_ | 98.3 |

**Table S4A: The bond distance for Zn^2+^-N^ε2^ of Ca^2+^-loaded CP* His_6_ site.**

|  |  | Residue | (A^o^) |
| --- | --- | --- | --- |
| H87C | S100A8 | Zn-N^ε2^_H20_ | 1.9 |
|  |  | Zn-O^2^_D30_ | 1.7 |
|  | S100A9 | Zn-N^ε2^_H83_ | 2.3 |
|  |  | Zn-S^ϒ^_C87_ | 2.5 |

**Table S4B: The bond angle for N^ε2^_x_-Zn^2+^-N^ε2^_y_ of Ca^2+^-loaded CP* His_6_ site.**

|  | Residue | Zn (^o^) |
| --- | --- | --- |
| H87C | N^ε2^_H20_-Zn-N^ε2^_H83_ | 104.6 |
|  | N^ε2^_H20_-Zn-S^ϒ^_C87_ | 112.6 |
|  | N^ε2^_H20_-Zn-O^2^_D30_ | 106.6 |

**Figure S1. Changes in the autocorrelation function for monodisperse CP* and CP* G102Stop upon the addition of Zn^2+^.** The addition of 0.7 equivalents of Zn^2+^ to a monodisperse solution of CP* and CP* G102Stop resulted in a significant shift in the autocorrelation function and an inability to fit the data to any of the standard models.


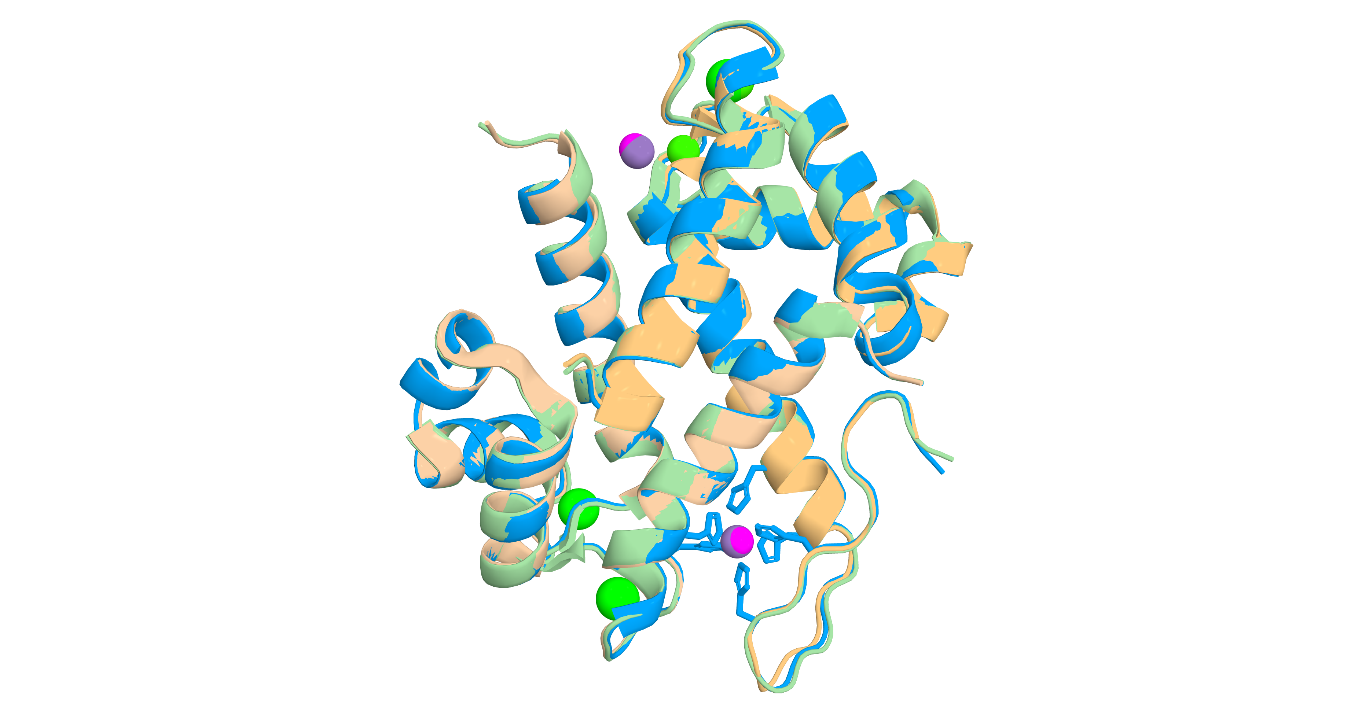


**Fig. S2. Comparison of CP crystal structures.** Overlays of the structures of Zn^2+^-CP* (dark green), CP H87C/ΔHis_6_ (blue), Ca^2+^-CP* (PDB-1XK4-orange), Ca^2+^+Mn^2+^-CP WT* (PDB-4GGF-green), and Ca^2+^+Ni^2+^-CP* (PDB-6DS2-yellow). Conformational changes induced by the binding of zinc are very modest.


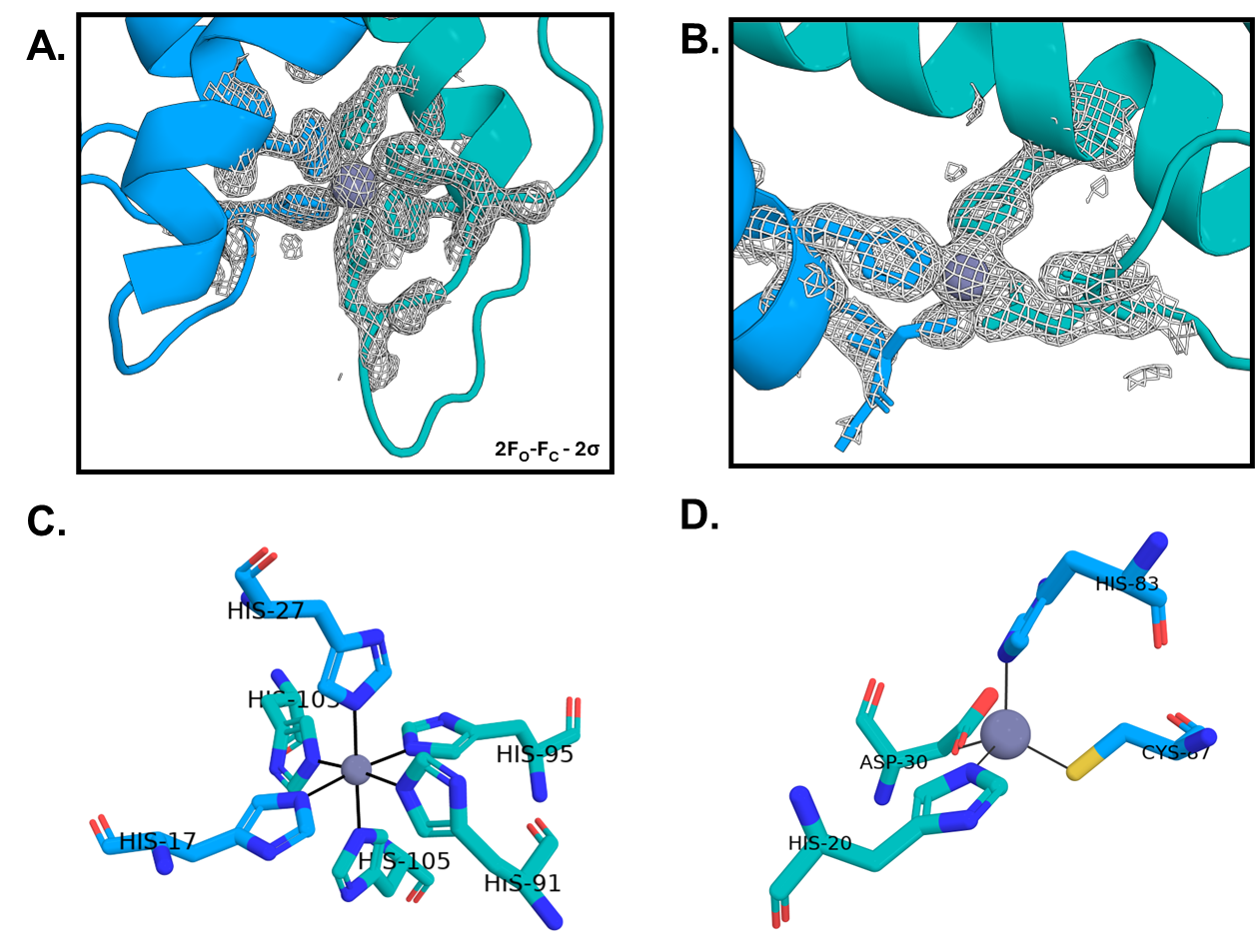


**Fig. S3. Coordination of Zn^2+^ ions by CP.** Electron density (2F_O_-F_C_ map contoured at 2σ) for the Zn^2+^ ions and coordinating residues of (A) Ca^2+^-loaded CP* and (B) Ca^2+^-loaded CP* H87C ΔHis_6_. (C) Octahedral coordination of a Zn²⁺ ion at the His₆ site Ca^2+^-loaded CP*. The Zn² ion (gray sphere) is coordinated by six histidine residues: His17 and His27 from S100A8, and His91, His95, His103, and His105 from S100A9. This hexavalent binding configuration is unique to CP and underlies its ability to bind a broad range of transition metals with exceptionally high affinity. (D) Tetrahedral coordination of a Zn²⁺ ion at the canonical His₃Asp site of Ca^2+^-loaded CP*, featuring a His87Cys mutation. The metal ion (gray sphere) is coordinated by His20, Asp30, and His83 from S100A8, along with Cys87 (substituted for His87).


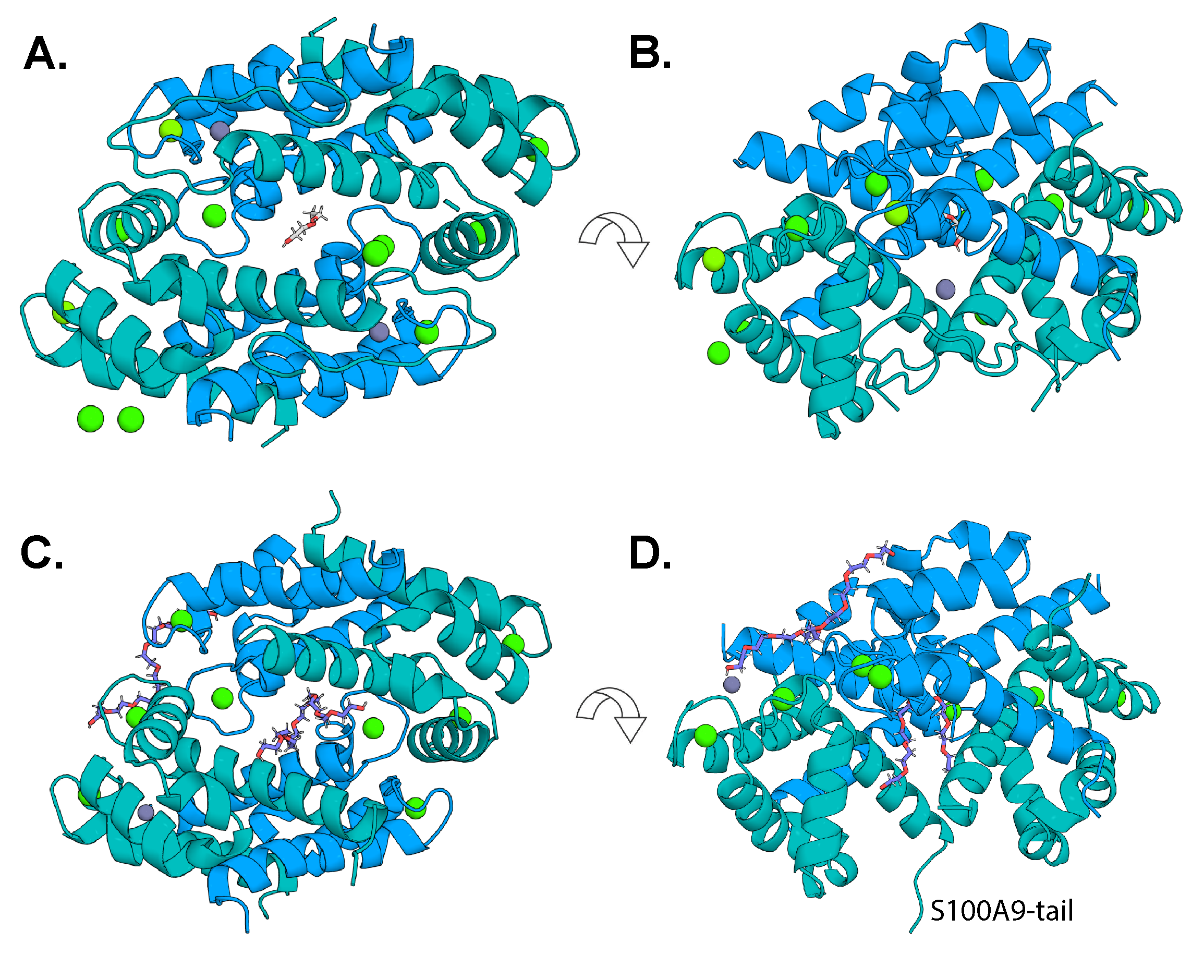


**Fig. S4. X-ray crystal structures of the Zn^2+^-bound Ca^2+^-loaded CP.** (A and B) The crystal structure of Zn^2+^-bound Ca^2+^-loaded CP* heterotetramer. (C and D) The crystal structure of Zn^2+^-bound Ca^2+^-loaded CP* H87C heterotetramer. Ca^2+^ ions are represented as green spheres, while Zn^2+^ ions as grey spheres. Note in panel D the observation of the S100A9 C-terminal tail, stabilized by crystal contacts, even though no ion is bound in His6 transition metal binding site.

**
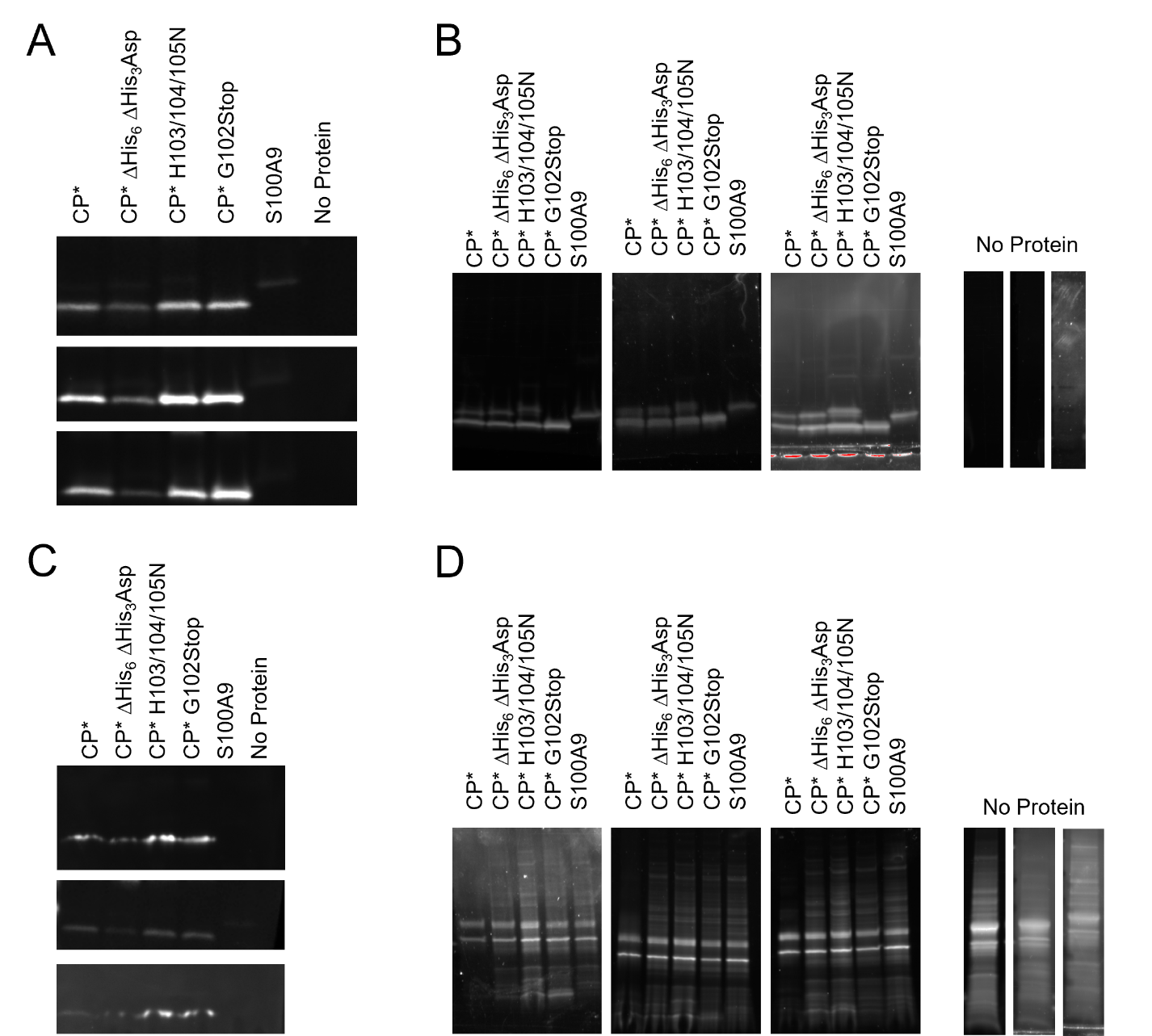
**

**Fig S5. Replicate images of immunoblots and Sypro Ruby stained gels are consistent with presented comparisons.** Replicate immunoblots staining for CP* and variants pre-incubation (A) and post-incubation (C) with *S. aureus*. Corresponding Sypro Ruby stained gels showing total protein pre-incubation (B) and post-incubation (D) with *S. aureus*.
